# Transfer learning by feature-space transformation: A method for Hippocampus segmentation across scanners^☆^

**DOI:** 10.1016/j.nicl.2018.08.005

**Published:** 2018-08-14

**Authors:** Annegreet van Opbroek, Hakim C. Achterberg, Meike W. Vernooij, M. Arfan Ikram, Marleen de Bruijne

**Affiliations:** a Biomedical Imaging Group Rotterdam, Department of Medical Informatics and Radiology, Erasmus MC - University Medical Center Rotterdam, 3000, CA, Rotterdam, the Netherlands; b Department of Radiology and Epidemiology, Erasmus MC - University Medical Center Rotterdam, Postbus 2040, 3000, CA, Rotterdam, the Netherlands; c Department of Computer Science, University of Copenhagen, DK-2100 Copenhagen, Denmark

**Keywords:** Classification, Domain adaptation, Hippocampus, MRI, Segmentation, Transfer learning

## Abstract

Many successful approaches in MR brain segmentation use supervised voxel classification, which requires manually labeled training images that are representative of the test images to segment. However, the performance of such methods often deteriorates if training and test images are acquired with different scanners or scanning parameters, since this leads to differences in feature representations between training and test data.

In this paper we propose a feature-space transformation (FST) to overcome such differences in feature representations. The proposed FST is derived from unlabeled images of a subject that was scanned with both the source and the target scan protocol. After an affine registration, these images give a mapping between source and target voxels in the feature space. This mapping is then used to map all training samples to the feature representation of the test samples.

We evaluated the benefit of the proposed FST on hippocampus segmentation. Experiments were performed on two datasets: one with relatively small differences between training and test images and one with large differences. In both cases, the FST significantly improved the performance compared to using only image normalization. Additionally, we showed that our FST can be used to improve the performance of a state-of-the-art patch-based-atlas-fusion technique in case of large differences between scanners.

## Highlights

• We present a feature-space transformation for image segmentation across scanners.
• This FST is trained on unlabeled images of subjects scanned with multiple scanners.
• These are used to transform training samples to values observed in target samples.
• The FST makes SVM hippocampus segmentation across scanners significantly better.
• Our FST can also increase performance of patch-based fusion methods.

## Graphical abstract

**Figure:**
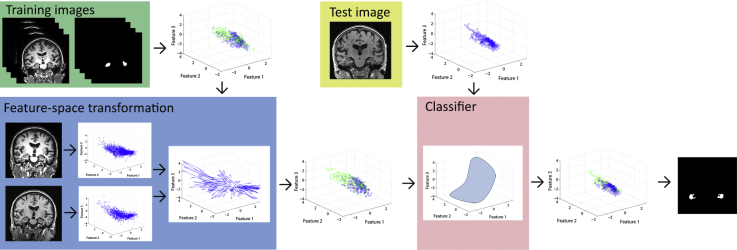

## 1 Introduction

The segmentation of medical images gives quantitative information about the tissues and structures of interest, which can aid both research and clinical diagnosis. Compared to manual segmentation, automatic segmentation can save large amounts of time and eliminate the problem of inter- and intra-observer variability. A widely used and successful method to perform such segmentations is by voxelwise classification based on supervised learning. Here, a manually annotated training set is used to extract features and train a classification system in the determined feature space. Then, the same features are determined for the test voxels and the trained classifier is used to make a decision on which label they should receive. Supervised-learning methods are used for a variety of segmentations tasks, such as whole brain (also called skull stripping) (Iglesias et al. (2011)), brain tissue (Mendrik et al. (2015)), white matter lesion (De Boer et al. (2009); Geremia et al. (2011)), and, combined with atlas registration, for segmentation of brain structures such as the hippocampus and cerebellum (Van der Lijn et al. (2012); Dill et al. (2015)).

Supervised-learning methods can perform very well if they are provided with a large enough training set that is representative of the test dataset. However, performance often deteriorates if training and test datasets have differences in appearance, which can lead to differences in sample distributions in the feature space that is used for the classification. These problems often happen because of differences between scanners or scanning parameters, for example in multi-center datasets. The most common way to deal with such differences between training and test data is by intensity normalization. Many methods in neuro-image segmentation use range matching, matching the mean and standard deviation of the datasets, or more extensive normalization techniques. Such extensive normalization techniques can roughly be separated into two groups, where the first group of methods first identify a tissue or multiple tissues of interest (such as white matter, gray matter, CSF, or background) in both the source and the target images and then match the peaks of these tissues in the intensity distributions (Schmidt (2005); Christensen (2003); Robitaille et al. (2012); Leung et al. (2010)). The second group of intensity-normalization techniques aim to match the intensity distributions of training and test images as a whole, without information of the imaged tissues (Nyul et al. (2000); Jager and Hornegger (2009); Weisenfeld and Warfteld (2004); Guimond et al. (2001)). The method of Nyul et al. (2000) is most widely used in neuro-image segmentation. It is shown to improve performance on e.g. brain-tissue and white-matter-lesion segmentation, both on same-scanner images (Zhuge and Udupa (2009)) and between scanners (Shah et al. (2011)).

However, image normalization techniques have the disadvantage that they aim at normalizing the image intensity only, while classification methods are often also based on other image-derived features. On the other hand, extracting derived features such as Gaussian-scale-space features from intensity-normalized images may still lead to different representations between scan protocols. Images are normalized by different mappings, which propagate differently in the derived features. In this paper, we propose a method that maps not only the intensity of training and test images, but also all the other features used for the classification, all at the same time. We will call this mapping a *feature-space transformation* (FST), since it maps the entire feature space of a training image to that of a test image. Our method learns the feature-space transformation from images of subjects that were scanned with both the training and the test scan protocol. Since our method involves learning, it can be called a *transfer-learning* technique (Pan and Yang (2010)). Transfer learning (sometimes also called *domain adaptation*) is recently gaining attention in medical image segmentation, since it aims to build a robust classification system by somehow compensating for differences between the distributions of training and test data. Van Opbroek et al. (2015b) showed that transfer-learning techniques can improve segmentation performance across scan protocols over intensity-normalization techniques such as range matching and the method of Nyul et al. (2000) in brain-tissue segmentation and white-matter-lesion segmentation.

A few papers have been published that apply transfer-learning techniques to neuro-image segmentation (Van Opbroek et al., 2015b, Van Opbroek et al., 2015c; Goetz et al. (2015); Kamnitsas et al., 2017; Van Tulder and De Bruijne (2016)). Most methods aim to compensate for the difference between training and test data in the classifier, for example with a weighted classifier that weights training samples according to resemblance to the test data. These methods show to improve performance compared to traditional, unweighted, classifiers when training and test data are from different scanners or scan protocols. However, these methods have the disadvantage that they only select samples as is, rather than learning how to transform the distribution of the training samples in the feature space as to better match the distribution of test samples. Some deep-learning methods take a different approach to transfer learning by learning a representation that is shared between data different scanners or scan protocols (Kamnitsas et al. (2017); Van Tulder and De Bruijne (2016)). This way, these methods learn a feature representation that is dataset invariant. We propose an approach for non-deep learning that, rather than learning a shared representation, maps the feature distribution of training samples directly to that of test samples. Our method learns an FST based on pairs of unlabeled images of one or multiple subjects that were scanned with both the source and target protocol. After transformation, a regular (non-transfer) classifier can be trained on the transformed features of the training data.

We performed a set of experiments on hippocampus segmentation in two heterogeneous datasets to show the added value of our FST over standard intensity normalization. Hippocampus segmentation is known to be a challenging task, since the gray levels of the hippocampus are very similar to those of neighboring structures such as the amygdala, thalamus, and caudate nucleus (Fischl et al. (2002)). Most hippocampus-segmentation methods are based on multi-atlas registration, where a set of training images (called *atlases*) are registered to the test image. The registered training images are then combined to obtain a final segmentation by an atlas-combination method such as majority voting or STAPLE (Warfield et al. (2004)). Performance can be greatly improved by combining registered atlases with appearance information such as voxel intensities in a supervised classifier (e.g. Powell et al. (2008); Coupé et al. (2011); Van der Lijn et al. (2012); Zhang et al. (2012); Wang et al. (2013)). However, incorporating such appearance information is likely to lead to problems when training and test images are obtained with different scan protocols. To decrease difference between training and test data, Van der Lijn et al. (2012) used intensity normalization to zero mean, unit norm and Coupé et al. (2011) used the technique of Nyul et al. (2000). In this paper, we investigate whether the use of an FST in such algorithms could improve performance over intensity-normalization techniques.

A preliminary version of this work has been published as a workshop paper (Van Opbroek et al. (2015a)). The present paper extends this workshop paper by thorough experiments on a new, enlarged version of the dataset presented in the workshop paper, one additional dataset, and comparison to STAPLE (Warfield et al. (2004)) and the patch-based-atlas-fusion method of Wang et al. (2013).

## 2 Material and methods

This section describes the proposed method and the data used in the experiments. The presented feature-space transformation is described in Section 2.1; Section 2.2 describes how the feature-space transformation is used in a voxel classifier; Section 2.3 presents the two datasets used in the experiments; and Section 2.4 describes the setup of the experiments.

### 2.1 Feature-space transformation

**Fig. 1:**
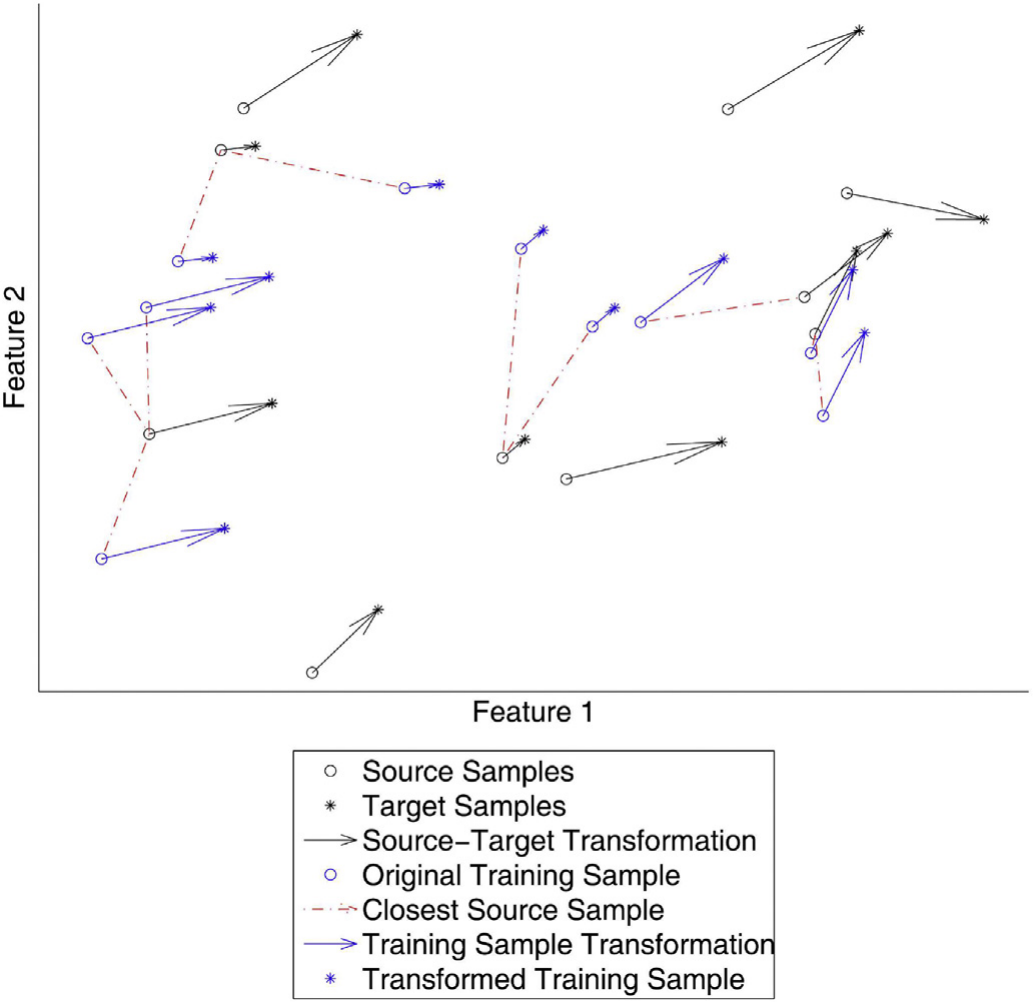
Schematic picture of the presented FST. Unlabeled source-target samples (shown in black) are generated from images of a subject scanned with both the source and target scanner. Labeled training samples (shown in blue) are then linked to their closest *k* source samples (here, *k* = 1, shown in red) and given the median transformation of these *k* source samples, which results in transformed training samples. (For interpretation of the references to color in this figure legend, the reader is referred to the web version of this article.)

We determine a feature-space transformation (FST) based on unlabeled images of subjects scanned with both source and target scanner. Here, the target image is affinely registered to the source image in order to obtain correspondences from each source voxel to a target voxel. Next, features are measured for the source and target voxels (from the original, unregistered images), so that the voxel correspondences become mappings in feature space. Finally, these voxel mappings are used to transform the feature values of the voxels from labeled training images to values observed in test voxels, as described in Section 2.1.2. Fig. 1 shows a schematic picture of the FST.

The FST therefore distinguishes two groups of samples. The first group consists of unlabeled source samples and target samples. These source and target samples originate from images of one or multiple subjects that have been scanned with both the source and the target scanner. The second group consists of training and test samples, where the labels of the training samples are used in a classifier to label the test samples.

#### 2.1.1 Notation

We first define the training and test samples. Let **x**_*i*_^*s*^ ∈ ℝ^*d*_*s*_^ denote a training sample consisting of a *d*_*s*_-dimensional feature vector at voxel *i* from the source scanner *s* and *y*_*i*_^*s*^ ∈ ℤ its label. Similarly, **x**_*i*_^*t*^ ∈ ℝ^*d*_*t*_^ denotes a *d*_*t*_-dimensional feature vector of a test sample in an image from the target scanner *t* and *y*_*i*_^*t*^ ∈ ℤ its label. Note that images from different scanners need not have the same number of features. All features need to be normalized (for example by a z-score transformation), so that distances calculated in feature space do not give different weights to differently scaled features.

All samples (voxels) follow a probability density function (PDF) in the feature space. We assume that samples from images that originate from the same scanner have similar PDFs and samples that originate from different scanners have different PDFs. We therefore distinguish between a source PDF and a target PDF.

The goal of our method is to determine an FST from the domain of *s*, *X*_*s*_ to the domain of *t*, *X*_*t*_:

(1) $$f_{s\to t}:X_{s}\to X_{t}.$$

*f*_*s*→*t*_ transforms the training samples **x**_*i*_^*s*^ from the source PDF to the target PDF by setting them to

(2) $${\tilde{x}}_{i}^{s}=f_{s\to t}\left(x_{i}^{s}\right).$$

The FST between a source scanner *s* and the target scanner *t* is learned from source and target samples: unlabeled voxels from images of *N* subjects that were scanned with both *s* and *t*. We call two images from the same subject obtained with *s* and *t* a *source-target pair*. These pairs should be acquired within a short time interval, so that the subject's anatomy can be assumed unchanged. Let **z**_*i*_^*s*^ ∈ ℝ^*d*_*s*_^ denote sample *i* from the source image of the source-target pair and **z**_*j*_^*t*^ ∈ ℝ^*d*_*t*_^ sample *j* from the target image of the source-target pair.

#### 2.1.2 FST determination

The target images of every source-target pair are affinely registered to their corresponding source images. A nearest-neighbor interpolation of the target images then provides a voxelwise correspondence for every sample **z**_*i*_^*s*^ to a sample **z**_*l*_^*t*^:

(3) $$\forall i:\exists \ell :z_{i}^{s}\to z_{\ell }^{t}.$$

For each training sample **x**_*i*_^*s*^, we determine the closest *k* source samples {**z**_*c*_1_^*i*^_^*s*^, **z**_*c*_2_^*i*^_^*s*^, …, **z**_*c*_*k*_^*i*^_^*s*^} in feature space, where *c*_*k*_^*i*^ denotes the *k*th closest sample to training sample number *i*. The FST of **x**_*i*_^*s*^ equals the transformation to the robust median target sample of these *k* source samples:

(4) $$f_{s\to t}\left(x_{i}^{s}\right)=x_{i}^{s}+\text{median}\left(z_{\ell_{1}}^{t}-z_{c_{1}}^{s},\ldots ,z_{\ell_{k}}^{t}-z_{c_{k}}^{s}\right),$$

where **z**_ℓ_*n*__^*t*^ (*n* = 1, 2, …, *k*) is the paired voxel of **z**_*c*_*n*__^*s*^ as defined in Eq. 3. The robust median of transformation vectors ***v***_1_, ***v***_2_, …, ***v***_*k*_ is defined as the transformation vector ***v***_*i*_ that has minimal total distance to all *k* transformations:

(5) $$\text{median}\left(v_{1},v_{2},\ldots ,v_{k}\right)=\arg\min_{v_{i}}\sum_{j=1}^{k}|v_{j}-v_{i}|.$$

We used the median (rather than the mean) to assure that the chosen transformation is one that is observed in the correspondence in Eq. 3.

Say that **z**_ℓ_*p*__^*t*^ − **z**_*c*_*p*__^*s*^ is the median transformation for **x**_*i*_^*s*^. Note that **x**_*i*_^*s*^ − **z**_*c*_*p*__^*s*^ is supposed to be small, since these points are close in feature space. Therefore *f*_*s*→*t*_(**x**_*i*_^*s*^) ≈ **z**_ℓ_*p*__^*t*^, so *f*_*s*→*t*_(**x**_*i*_^*s*^) is approximately distributed by the same distribution as **z**_ℓ_*p*__^*t*^, the target distribution.

Higher *k* increases the regularization, which results in a smoother transformation. In our experiments where we compared the presented FST with other methods, we used *k* = 1 as default. In some extra experiments we showed the effect of increasing *k*.

In our experiments, we train only on images from a single source scanner. However, when one has training datasets from multiple scanners, each dataset could be transformed to the test dataset individually if source and target images are available on each source scanner and the target scanner.

### 2.2 Hippocampus segmentation

**Fig. 2:**
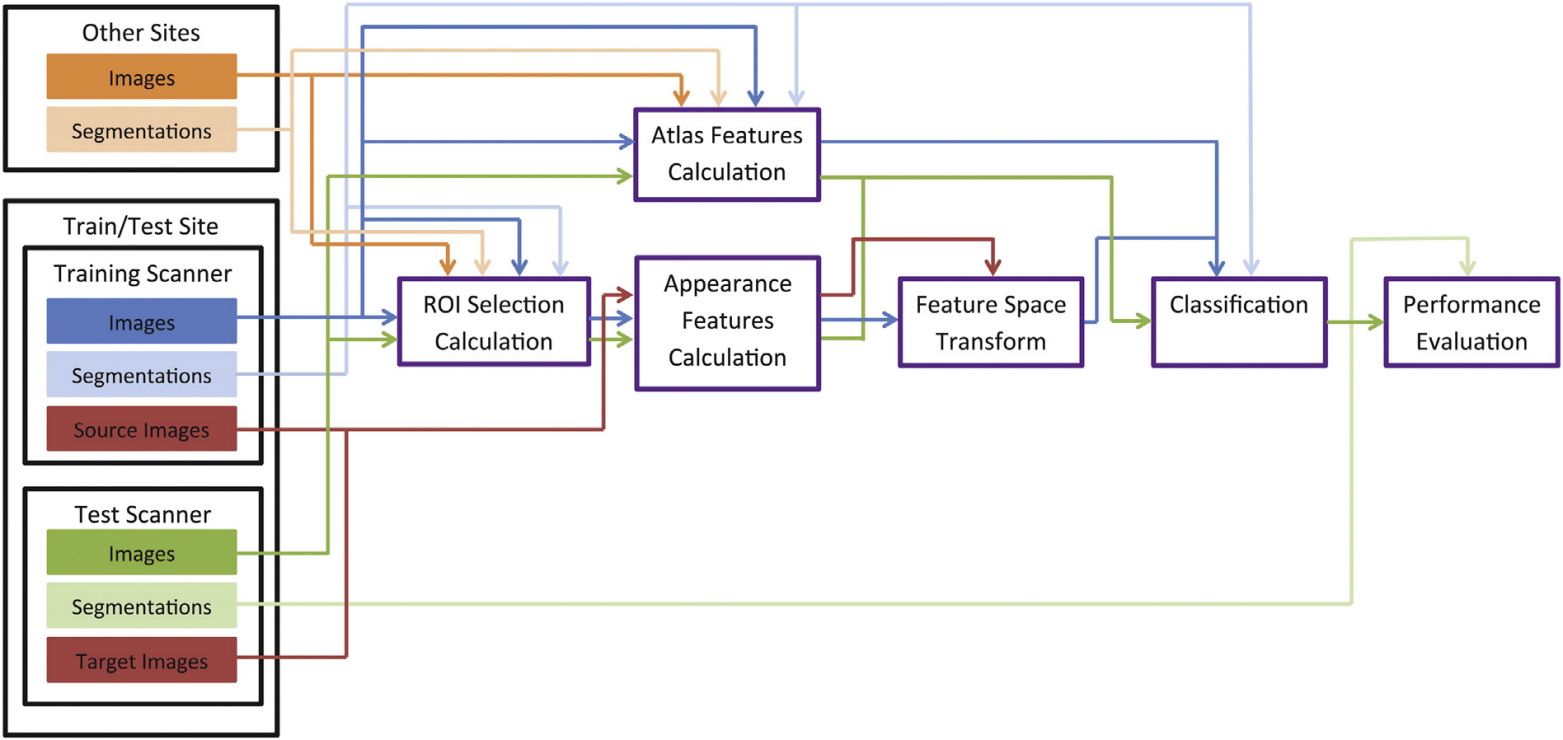
Overview of the presented method (best viewed in color). The used data is on the left, the different steps of the method are depicted from left to right. Methods are in purple, where data that is used in the method goes in from the top, processed data flows in from the left and out from the right. Source and target images are images from the same subject. For the HarP experiments, there are 8 train/test sites, categorized in Table 1. For the RSS experiments, there is only one train/test site with 20 images from one scanner and 18 from the other. Data from “other sites” is used in the HarP2 experiments only and consists of 33 sites (135 images minus the number of images in the train/test site, given in Table 1). (For interpretation of the references to color in this figure legend, the reader is referred to the web version of this article.)

The hippocampus segmentation was performed by voxelwise classification within a region of interest (ROI) around the hippocampus. Fig. 2 gives an overview of the data and the steps for the presented method.

#### 2.2.1 Multi-atlas probability

A training set of atlases (where the hippocampi have value one and non-hippocampi has value zero) was used to determine a probability per training and test voxel of it being hippocampus. For the RSS and HarP 1 experiments (specified in Section 2.4.1), the training atlases were the same as the training images. For HarP2 and HarP3, all training atlases were non-rigidly registered to the test images and to each other as described in Section 2.3.4. The multi-atlas probability per test voxel was then determined by averaging the values of all registered training atlases. Similarly, the multi-atlas probability for the training images was determined by averaging the values of all registered training atlases.

The ROI was determined as all voxels with a multi-atlas prior probability of at least 10%. This threshold was chosen manually in a trade-off between accuracy and speed as to exclude as many non-hippocampus voxels and as few hippocampus voxels as possible.

#### 2.2.2 Features

The multi-atlas probability was used as a feature in the classifier. Additionally, 10 local image-appearance features were used:

• the voxel intensity
• the intensity after a Gaussian smoothing at *σ* = 1, 2.2, and 5 mm^3^
• the gradient magnitude after a Gaussian smoothing at *σ* = 1, 2.2, and 5 mm^3^
• the Laplacian after a Gaussian smoothing at *σ* = 1, 2.2, and 5 mm^3^

These features are a subset, consisting of all rotationally invariant features of those used by Van der Lijn et al. (2012) for hippocampus segmentation. Only the rotationally invariant features were chosen in order to cope with differences in patient orientation.

The appearance features were normalized per scanner to zero mean, unit variance within the brain mask. The multi-atlas probability was normalized to zero mean, unit variance based on the samples within the ROI around the hippocampus, since this feature is mostly zero outside the ROI.

The appearance features of the training samples were transformed with the FST. The multi-atlas prior probability was not transformed, as this feature was assumed to not be influenced by the scanner appearance differences.

#### 2.2.3 Classification

The segmentation was obtained by voxelwise classification with a support vector machine (SVM) (Cortes and Vapnik (1995)) with a Gaussian kernel (Scholkopf and Smola (2001)). The SVM was trained on a uniformly randomly selected subset of samples inside the ROI of the training images. After training, the SVM was applied to all test samples within the ROI of the test images.

### 2.3 Data

We present results on two datasets. The first dataset consists of ADNI (Mueller et al. (2005)) data, which has been acquired with various scanners with similar scanning protocols. The second dataset consists of Rotterdam Scan Study (Ikram et al. (2015)) data, which has been acquired with two scanners with different scanning protocols. As a result, in the first dataset the differences in appearance between images from different scanners are much smaller than is the case in the second dataset.

#### 2.3.1 Dataset1: Harmonized protocol

The first dataset consists of Harmonized Protocol (HarP) data^1^. This dataset consists of 135 Alzheimer's Disease Neuroimaging Initiative (ADNI) T1-weighted images (Mueller et al. (2005))^2^ with manual hippocampus segmentations (Boccardi et al. (2015)). These 135 images were scanned at 34 sites, of which 12 sites scanned subjects with both a 1.5 T and a 3 T scanner. For 8 of these 12 sites we found pairs of (unlabeled) images in the ADNI database^3^ of subjects that were scanned with both the 1.5 T and the 3 T scanner within a month from each other. The 45 HarP images and segmentations of these 8 sites were used as training and test data, where each image was segmented by training on all images from the other scanner at the same site. A maximum of four pairs of the unlabeled ADNI images per site were selected to be used as source-target pairs to determine the FST between the scanners. Table 1 gives the number of images per site and per scanner in the HarP dataset.

**Table 1:** Subjects per site in the HarP datasets that were included in the training and test sets.

Site number	Number of images	
	1.5T	3T
002	7	7
005	3	2
007	2	2
013	3	2
016	3	3
020	1	1
126	1	2
127	2	4
Total	22	23

**Fig. 3:**
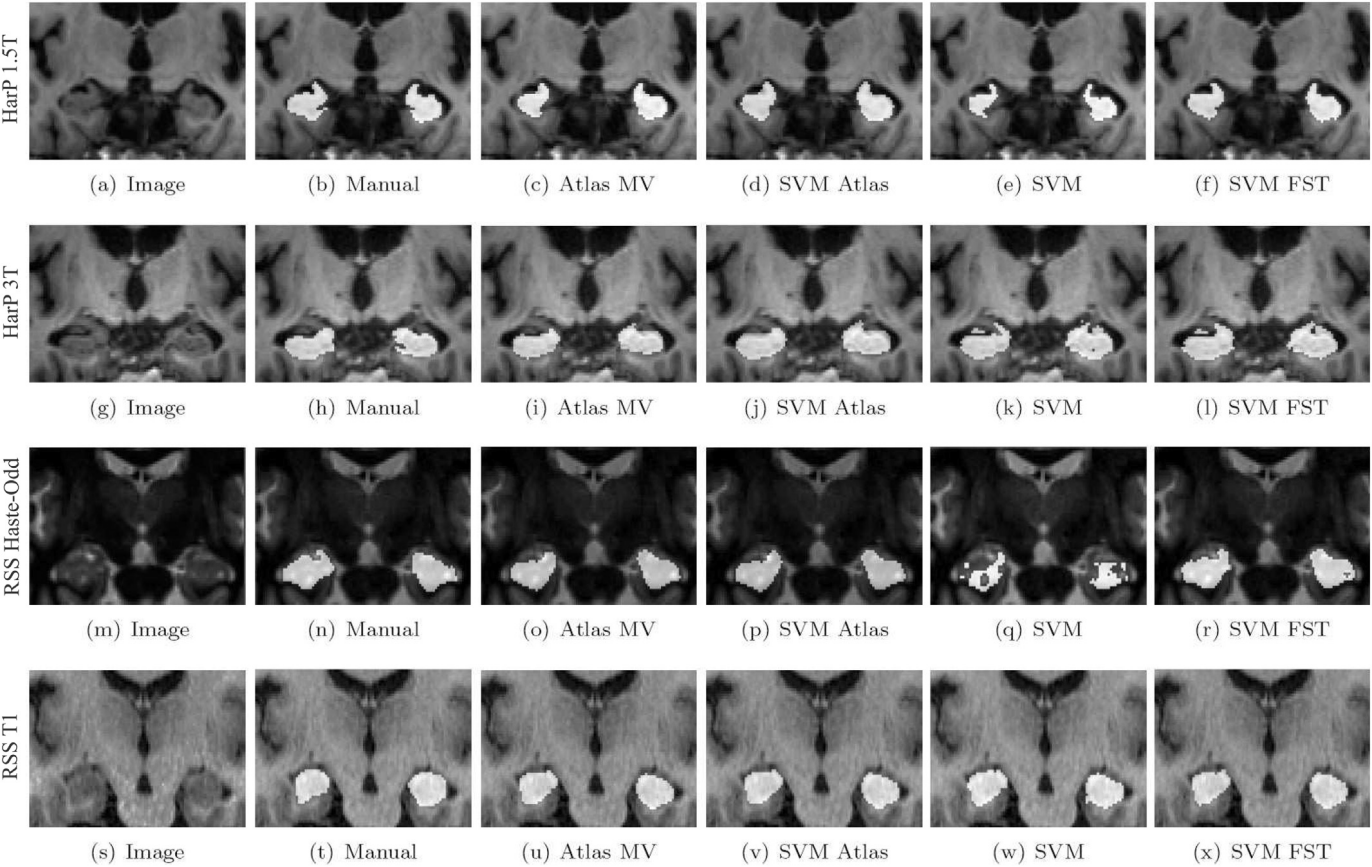
Example hippocampus segmentations for the various methods overlaid on the bias-field corrected images. (a)–(f): HarP dataset with all different-scanner images used as atlas, 1.5T images segmented by training on 3T images and (g)–(l): 3T images segmented by training on 1.5T images. (m)–(r): RSS dataset, Haste-Odd images segmented by training on T1 images and (s)–(x): T1 images segmented by training on Haste-Odd images. Examples were chosen to have Dice overlap as close as possible to the mean Dice overlap on all images.

Fig. 3(a), (g) give an impression of the difference between a 1.5 T and a 3 T scan from the same site and Fig. 3(b), (h) of their manual segmentation.

#### 2.3.2 Dataset 2: Rotterdam scan study

The second dataset consists of images of healthy elderly volunteers from the Rotterdam Scan Study (RSS) (Ikram et al. (2015)) with manual hippocampus segmentations. 20 images were obtained with a 1.5 T Siemens scanner with a Haste-Odd protocol (inversion time = 4400 ms, TR = 2800 ms, TE = 29 ms)(Ikram et al. (2008)); 18 images were obtained with a 1.5 T GE scanner with a T1 protocol (Ikram et al. (2015)). The datasets were segmented by different observers.

As source-target pairs, we used rescan images of 9 subjects that were scanned with both scanners within a short time interval from each other. Fig. 3(m), (s) show an example Haste-Odd image and T1 image and Fig. 3(n), (t) show their manual hippocampus segmentations.

#### 2.3.3 Preprocessing

All images were rigidly registered to MNI152 space with 1 × 1 × 1 mm^3^ voxel size as described in Section 2.3.4 and corrected for MRI bias field with the N4 method (Tustison et al. (2010)). Next, a brain mask was determined as follows. For the HarP dataset, the brain extraction tool (BET) (Smith (2002)) was run with default parameters on all images. Since this gave variable results, a second step was applied. Here, the BET segmentations of all images were non-rigidly registered to each other and per image a majority vote was performed. For the RSS data, a slightly different approach was used since BET gave bad results for the Haste-Odd images. Here, BET was run with default parameters on the 9 T1 rescan images. These masks were then transformed to the Haste-Odd rescan images by an affine registration of each T1 rescan image to its corresponding Haste-Odd rescan image. The final brain masks for all images (also the rescan images) were obtained by non-rigid registration of the 9 rescan images of the same scanner followed by a majority vote.

Before calculation of the appearance features, all images were normalized for intensity by a 4th–96th percentile range matching procedure within the brain mask.

#### 2.3.4 Registration

All registrations were performed with the Elastix registration toolbox (Klein et al. (2010)) based on maximizing normalized mutual information. We used the registration settings of Bron et al. (2014), which were visually optimized for ADNI data.

The source-target pairs were registered to each other by a rigid registration followed by an affine registration, to compensate for possible distortion. The brain masks were registered by consecutively running a rigid, affine, and non-rigid registration. The multi-atlas probabilities were obtained by an initial rigid registration of the brain masks, followed by a rigid, affine, and non-rigid registration of the images, where only voxels inside the brain masks contributed to the similarity measure.

### 2.4 Experimental setup

#### 2.4.1 Experiments

From the source and target images, we used all voxels within the brain mask to determine the FST. Next, the training voxels inside the ROI were transformed with the FST and used to train an SVM classifier. Finally, all voxels inside the ROI of the test images were classified as hippocampus or non-hippocampus.

For both the HarP and the RSS dataset, we segmented each image by training on all images scanned with the other scanner at the same site. For the RSS dataset, this means training on the GE scanner and testing on the Siemens scanner and vice versa. For the HarP dataset, we segment all 45 images from each of the 8 sites in Table 1 by training on the other scanner of the site, so training on 1.5 T and testing on 3 T and vice versa. We will refer to this experiment as HarP 1.

Additionally, we performed an extra experiment per dataset. As can be seen from Table 1, only between 1 and 7 training images could be used for HarP 1. Unfortunately, images from other sites could not be used, since no source-target pairs were available between sites. However, a possible way to improve the performance, which was also investigated, is by determining the multi-atlas-probability feature on all images from different scanners than the test image, which results in a total of 128 to 134 atlases (Fig. 2 including the “other sites”). This dataset will be referred to as HarP 2. For methods without FST (which were studied for comparison), we also performed an experiment where we train both the atlas features and the appearance features on both the training scanner and the images from other sites. This dataset is referred to as HarP3. Table 2 shows the differences between HarP1, HarP2, and HarP3.

**Table 2:** Data used in the different datasets.

Dataset	Train scanner		Other sites	
	Appearance	Atlas	Appearance	Atlas
HarP1	x	x		
HarP2	x	x		x
HarP3	x	x	x	x
RSS	x	x		

On the RSS dataset, we performed an additional experiment where we segmented the two rescan images of all 9 subjects in cross validation, by training the FST on one of the other rescan images. Here, both scanners were once used as training scanner to segment all rescan images; where we compared the difference in segmented volume between the two rescan images of all 9 subjects. This experiment was performed to study the influence of our method for the reproducibility of segmentations across scanners.

For both datasets, we also studied the influence of the number of source-target images, *N* and the number of neighbors, *k*, that were used in the FST. We also compared the performance of our SVM with FST to that of two established hippocampus-segmentation methods: STAPLE (Warfield et al. (2004)) and the multi-atlas label-fusion method of Wang and Yushkevich (2013).

The performance of the various methods was measured in terms of Dice overlap (Dice (1945)) between the resulting segmentations and the manual segmentations, averaged for left and right hippocampus. Significance of differences was determined with a Wilcoxon signed-rank test per subject, with the significance threshold at *P* = .05. The repeatability in *RSS Rescan* is shown in a Bland-Altman plot, which shows the difference in volume between the outputs of two methods as a function of the average volume of the two outputs.

#### 2.4.2 Compared methods

We compared the performance of the following methods:

*Atlas MV*: Majority vote, where the segmentation was obtained by thresholding the multi-atlas probability feature at 0.5.

*SVM Atlas*: The SVM classifier on just the multi-atlas probability feature. This method was added to determine how much of the difference in performance between the *SVM* method (below) and the Atlas MV method can be explained by the probability feature.

*SVM*: The SVM classifier on the multi-atlas probability and the appearance features, without the feature-space transformation.

*SVM FST*: The SVM classifier on the multi-atlas probability and the appearance features with the feature-space transformation.

*SVM FST*_*Intensity*_: Similar to SVM FST, but with the FST applied to the intensity feature only. The other features were calculated from the transformed intensity image. This method was added to show the added value of transforming all features at the same time over transforming intensity alone.

*SVM Image Weighting*: On the Harp datasets, we compared to the BD image-weighting method of Van Opbroek et al. (2015c), which is also designed to cope with images from different scanners. This method weights all training images according to PDF similarity with the test image. These weights are then used to select training samples, which are used to train an SVM classifier. This method was not applied to the RSS dataset because the differences between training and test data are too large for this method to cope with.

Additionally, we compared the performance with that of two state-of-the-art hippocampus-segmentation methods; one that uses only atlas information and one that incorporates atlas and appearance information:

*STAPLE*: Here, atlases were combined with the Simultaneous Truth And Performance Level Estimation (STAPLE) algorithm (Warfield et al. (2004)).

*Fusion*: The multi-atlas-label-fusion method of Wang and Yushkevich, 2013, without corrective learning (Wang et al. (2011)). This method won third place at the MICCAI 2012 Grand Challenge and Workshop on Multi-Atlas Labeling on hippocampus segmentation.

We also investigated a combination of our FST and *Fusion*:

*Fusion FST*_*Intensity*_: Here, the FST was determined in the feature space used for *SVM FST* in order to transform image intensities of the training data to those observed in the test data. The transformed intensity images were subsequently used for the patch-based fusion. Here, only the intensity was transformed, contrary to all features, because we used a readily available implementation of Fusion, which does not allow the transformation of all features.

Note that *SVM FST*, *SVM FST*_*Intensity*_, and *Fusion FST*_*Intensity*_ could not be applied to the HarP3 dataset, since this would require every training image to be transformed (also from the “other sites”), which is not possible because source-target pairs were not available between all training and test images.

#### 2.4.3 Implementation and parameters

For all SVM classifiers, we used LIBSVM (Chang and Lin (2011)). For STAPLE the CRKIT^4^ was used. For *Fusion* we used the implementation of the authors of the paper (Wang and Yushkevich (2013))^5^. For both *STAPLE* and *Fusion* the default parameters were used.

All SVM classifiers were trained on 10,000 training samples. This number was chosen on a subset, as a trade-off between accuracy and computation time. The SVM slack parameter *C* and the kernel parameter *γ* were determined in cross validation on the training set. Here, for the HarP dataset leave-one-site-out cross validation was used. This way, the parameters were optimized to cope with differences between images from different sites. For the RSS dataset, leave-one-image-out cross validation was used, since there were not enough different sites (or scanners) in this dataset to perform leave-one-site-out cross validation.

Separate classifiers were trained for the left and right hippocampus, which improved performance compared to training a single classifier. Probably, this is because the left and right hippocampus have slightly different appearance.

The sample correspondence for the FST in Eq. 3 was determined on all voxels within the intersection of the source and target brain masks. For the FST, we used *k* = 1 number of neighbors and *N* = max (*N* = 4 for HarP data and *N* = 9 for RSS data) number of source-target image pairs. In Section 3.5, we investigate the influence of *k* and *N*.

## 3 Results

### 3.1 Comparison of appearance features with and without FST

Table 3 shows the mean performance for *Atlas MV*, *SVM Atlas*, *SVM*, *SVM FST*, *SVM FST*_*Intensity*_, and *SVM Image Weighting* on 1) the HarP1 dataset, 2) the HarP2 dataset, with additional “other sites” used as atlas, and 3) the RSS dataset. For all three cases, training an SVM on only the multi-atlas probability (*SVM Atlas*) improved the performance over setting the multi-atlas threshold at 0.5 (*Atlas MV*). Adding appearance features (without FST), as in the *SVM* method, improved performance only in HarP 1. This overall decrease in performance is because appearance differs between the scanners and is therefore misleading for classification. In HarP 2, the performance decreased by adding appearance features without FST, probably because here better atlas information is available than in HarP 1. For the RSS dataset, where appearance differs much more between training and test images than in the HarP dataset, the appearance features harmed performance most. Adding appearance features with FST, as in the *SVM FST* method, significantly improved the performance over using only multi-atlas information (*SVM Atlas*) and using appearance features without FST (*SVM*) in all three cases.

**Table 3:** Mean Dice overlap of the various methods on 1) the HarP1 dataset trained on images from the same site other scanner than the test image, 2) the HarP2 dataset with multi-atlas probabilities determined from all HarP images except for the ones from the test scanner, 3) the RSS dataset. The best result and the results that were not statistically significantly worse, are shown in bold.

Method	HarP 1	HarP 2	RSS
Atlas MV	0.725	0.793	0.791
SVM Atlas	0.729	0.827	0.797
SVM	0.743	0.786	0.726
SVM FST	**0.753**	**0.840**	**0.804**
*SVM FST*_*Intensity*_	0.690	0.727	0.411
SVM Image Weighting	0.744	0.788	n.a.

Applying the FST only on the intensity feature and calculating the other appearance features from the transformed image, as in *SVM FST*_*Intensity*_, performed much worse than applying the FST to all appearance features in all three experiments. On RSS, this method performed especially bad, which might be caused by the large difference between training and test data. Transforming the intensity will overcome intensity differences, but this transformation is not smooth in image space. The appearance features calculated from the transformed intensity can then become very different from the features in the test image. The other transfer-learning method, *SVM Image Weighting*, performed only marginally better than *SVM*. This is probably because the method was designed to be trained on larger, more diverse, datasets. Here it was trained on only few training images, where it often gave a positive weight to only a single training image, which did not give a good classifier.

### 3.2 Comparison with state-of-the-art methods

Table 4 shows the performance of *SVM*, *SVM FST*, *STAPLE*, *Fusion*, and *Fusion FST*_*Intensity*_ on the HarP1, HarP3^6^, and RSS dataset. Note that for *STAPLE*, performance on HarP2 and HarP3 are the same, since this method uses no appearance features. For *Fusion* on the other hand, performance on HarP2 could not be calculated since this method can not use atlas information without appearance information.

**Table 4:** Mean Dice overlap of our method compared to state-of-the-art methods on 1) the HarP1 dataset trained on images from the same site other scanner than the test image, 2) the HarP3 dataset trained on all HarP images except for the ones from the test scanner, 3) the RSS dataset. The best result and the results that were not statistically significantly worse, are shown in bold. N.a. = not available; this method is not available since no source-target pairs were available between sites. For STAPLE, the results on HarP3 equal the results on HarP2, since it does not use appearance features.

Method	HarP 1	HarP 3	RSS
SVM	0.743	0.861	0.726
SVM FST	0.753	n.a.	0.804
STAPLE	0.718	0.827	0.799
Fusion	**0.798**	**0.884**	0.336
*Fusion FST*_*Intensity*_	0.773	n.a.	**0.816**

*STAPLE*, which uses only atlas information, performed similar to the other two methods that use only atlas information, *Atlas MV* and *SVM Atlas*. When appearance differences are small (the HarP datasets), *Fusion* greatly outperforms *STAPLE*, *SVM*, and *SVM FST*. *Fusion* therefore seems to use a framework that is better capable of handling small appearance differences than the baseline *SVM*. When differences are large however (the RSS dataset), performance of *Fusion* dropped dramatically (much more than *SVM*). Using an FST to transform image intensity before using Fusion, as in *Fusion FST*_*Intensity*_ greatly improved the performance in case of large differences. For small differences however, *FST*_*Intensity*_ decreased performance. We argue that this is probably because transforming only the intensity is a suboptimal solution, as was also shown in Table 3 for *SVM FST*_*Intensity*_. black.

### 3.3 Quantitative results

Fig. 3 shows example segmentations for *Atlas MV*, *SVM Atlas*, *SVM*, and *SVM FST* on the HarP 2 dataset and on the RSS dataset. For all four images, the methods that use only atlas information, *Atlas MV* and *SVM Atlas*, produced segmentations that are too smooth compared to the manual segmentations. The methods that combine atlas information and appearance information, *SVM* and *SVM FST* gave more detailed segmentations, where *SVM FST* gave the best segmentations. In Fig. 3(e) and 3(q) we can see that *SVM* produced an under segmentation because of the difference in appearance between training and test data. As can be seen from Fig. 3(f) and 3(r) this problem was solved by using the FST.

The example segmentations also show a disadvantage of adding appearance features: it increases the chance of obtaining a segmentation with incorrect topology (i.e. an unconnected segmentation or a segmentation with a hole). This may happen in voxel classifiers that incorporate appearance features because non-neighboring voxels in the image might be close to each other in the feature space, while neighboring voxels in the image are not necessarily close in the feature space. This can easily be solved by a post-processing step such as morphological opening, taking the biggest connected component, smoothing of posterior outputs in the image space, or a graph cut (Van der Lijn et al. (2012)).

### 3.4 RSS rescan segmentation

**Fig. 4:**
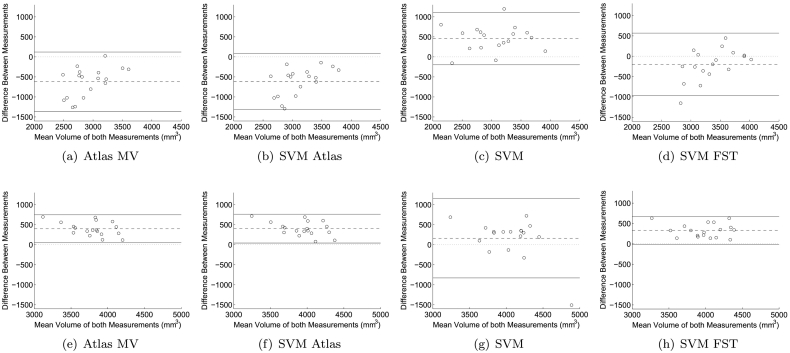
Bland-Altman plots for *Atlas MV*, *SVM Atlas*, *SVM*, and *SVM FST* (*N* = 8) in *RSS Rescan*: reproducibility on the hippocampus segmentation of 9 rescan images in the RSS dataset. (a)–(d): trained on T1 images, (e)–(h): trained on Haste-Odd images. Each sample is one hippocampus (left or right).

Fig. 4 shows Bland-Altman plots for *Atlas MV*, *SVM Atlas*, *SVM*, and *SVM FST* on segmenting the RSS rescan images. When training on the T1 images (Fig. 4(a)-(d)), *SVM FST* showed a much smaller bias than the other methods and similar variance, indicating more consistent segmentation results across scanners. When training on the Haste-Odd images, *Atlas MV*, *SVM Atlas*, and *SVM FST* showed similar bias and variance. *SVM* gave a smaller bias, but a much larger variance than the other methods.

Note that the mean volume was much larger when training on the Haste-Odd images than on the T1 images (4000 versus 3000 mm^3^). This is partly a result of the manual segmentations, which are on average about 15% bigger in the Haste-Odd images than in the T1 images and partly a result of the larger voxel sizes for the Haste-Odd images.

### 3.5 Influence of *k* and *N*

**Fig. 5:**
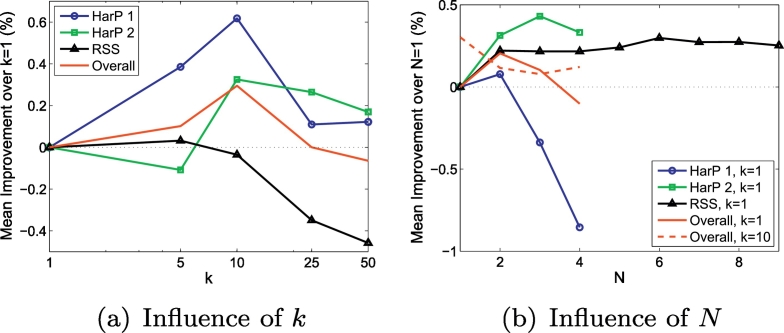
Influence on the performance of the number of neighbors, k and the number of source-target images, N. Figure (a) shows the influence of k for N = 1; Figure (b) shows the influence of N for k = 1 and k = 10 (only average over the three datasets). All results are shown as improvement in Dice over the performance with k = 1, N = 1.

Fig. 5 shows the influence of *k* and *N* on the performance of our FST for the two experiments on the HarP data and the experiment on the RSS data. As can be seen from the figure, the influence of both *k* and *N* on the performance is very small; the difference in average Dice between the worst and the best *k* and *N* is only 0.6%. *N* = 1, *k* = 10 seems the overall best choice, performing significantly better than the other values for *k* and non-significantly different from other values for *N*.

### 3.6 Computational cost

The computational time of the FST was in the order of minutes and depended on *N* and *k*. A source-target registration and sampling the voxels took only a couple of seconds. Determining for all training samples the closest *k* source samples in feature space was the most expensive operation, but can be efficiently computed with a *k*-d tree, which is $O\left(n\right)$ in worst case.

## 4 Conclusion and discussion

### 4.1 Conclusion

We presented a feature-space transformation (FST) to decrease appearance differences between training and test datasets caused by the use of different scanners or scanning parameters. Our method uses unlabeled images of one or multiple subjects that have been scanned with both the training and the test scan protocol. These images, which we call source-target pairs, give a correspondence between the source and target feature spaces. Training samples are then mapped from the source feature space into the target feature space by applying the median transformation of the *k* closest source voxels in the feature space.

We presented extensive experiments on hippocampus segmentation based on both appearance and atlas features in two datasets: one with relatively small differences between scanners and one with very large differences.In the first dataset, the presented FST improved the performance of an SVM classifier on atlas and appearance features from a mean Dice of 0.74 to 0.75 when few atlases were used and from 0.79 to 0.84 when many atlases were used. In the second dataset, our FST improved the mean Dice from 0.73 to 0.80. On this dataset, we also showed that the FST can be used in combination with patch-based atlas fusion to improve performance across scanners with large differences. Additionally, we showed that our FST can improve the reproducibility across scanners, by decreasing the bias between segmentations of images from different scanners.

We believe that the presented method is very useful for machine-learning based segmentation of medical images that have been obtained with different scanners or scanning protocols and have images of a subject acquired with both scanners. The experiments in this paper were all on hippocampus segmentation. However, the presented FST can be used for many more supervised image segmentation tasks, such as brain-tissue segmentation, white-matter lesion segmentation, and segmentation of other brain structures than the hippocampus. This way, we think that our method can aid the applicability of many supervised-segmentation methods to different datasets and eliminate the requirement of same-scanner labeled training data.

### 4.2 Comparison with other methods

We compared with multiple other methods for hippocampus segmentation: a multi-atlas registration with majority vote, atlas fusion by STAPLE (Warfield et al. (2004)), and the patch-based atlas fusion method of Wang et al. (2013). Majority vote and STAPLE make a decision based only on atlas information, while patch-based fusion methods, just like the used SVM classifier, incorporate appearance information, which overall performed better if appearance is similar. Patch-based atlas fusion outperformed the baseline SVM in case of small differences between train and test data, but decreased performance much more in case of large differences. We think patch-based atlas fusion is a better framework for atlas-based hippocampus segmentation than the baseline SVM, in case of training and test data from the same scanner, or when differences between images from different scanners are small. This can be explained by patch-based fusion makeing better use of the atlas information, by combining appearance information of every training sample (voxel) with the atlas prior of its image. The SVM combines the appearance information of training samples with the atlas prior of all images together. It therefore gives all atlases the same weight, while the patch-based fusion gives large weights only to the atlases with most similar appearance, which is beneficial if appearance is similar between train and test images. However, when appearance information is misleading, as in datasets with large differences between training and test data, patch-based fusion deteriorates more, because of this effect. We think that the presented FST can solve this problem, by transforming the representation of training samples to that of test samples. We also experimented with a poor man's implementation of such an FST for patch-based fusion, by transforming all training voxels in the feature space of the SVM FST, generating a transformed intensity image, and feeding this image into the patch-based fusion. This procedure greatly improved performance of patch-based fusion in the RSS dataset, but decreased performance on the HarP dataset. However, we think that performing an FST in the patch feature space used in patch-based fusion, rather than transforming only the intensity feature, would solve this problem. We namely showed for the SVM that transforming all features with the FST works much better than transforming only the training voxel's intensity.

### 4.3 Related work

Our approach is inspired by the patch-based image-synthesis techniques of e.g. Roy et al. (2011); Iglesias et al. (2013), which aim to make the appearance of source images similar to that of a target scanning protocol. These methods extract source-target patches from source and target scans of the same subject and then adapt the intensities of a new image by splitting it up into patches and determining the closest source patch. In contrast, our FST performs a transformation in the higher-dimensional feature space that is used for the classification.

Recently, methods have been developed to perform CT-MRI image synthesis with CycleGANs (Chartsias et al. (2017); Wolterink et al. (2017); Huo et al. (2018); Zhang et al. (2018)). Here, a convolutional neural network (CNN) is trained that consists of two competing components, which are iteratively optimized: a generator, which transforms images from one modality to the other, and a discriminator, which discriminates between generated images and real images from the target modality. This way, image synthesis can be trained on unpaired source-target images. Since paired images are not always available, we think it would be very interesting to investigate the use of such methods for neuro-image segmentation across MRI scanners and modalities.

### 4.4 Features used for FST

We compared transforming all features to an FST on the intensity feature only, followed by calculation of the other features from the transformed intensity image. An FST on all features clearly outperformed FST on intensity alone, because image features derived for a transformed intensity image often appear quite different from the (non-transformed) features in the test data, due to e.g. noise.

In the experiments, we focused on only intensity and Gaussian-scale-space features in cases where the same features are extracted for source and target data. However, the FST can also be applied to different features and to situations with differences between source and target features. We did not use spatial information in the FST, since this could result in only voxels around the hippocampus being used for the FST. We have in a preliminary stage experimented with learning the FST from the entire image or only from the ROI and found the former to give a much better FST, since it uses many more voxels. For segmentation purposes without a strong spatial prior, such as brain-tissue segmentation, it might be beneficial to include spatial features to help with possible spatial distortions that differ between train and test scanner, such as bias fields.

Also, unlabeled source-target images should be representative for the training data. Problems may arise if, training data contains tissues that are not observed in source-target data (such as tumors), for it would not be possible to learn the proper transformation. Lastly, there should be a one-to-one mapping between classes in source and target data in order to learn a good FST.

### 4.5 Limitations and future work

A limitation of the presented method is the requirement of same-subject images on both the training and the test scanner. In single-site studies such as the RSS (Ikram et al. (2015)), rescans are often made in order to check for reproducibility and to eliminate scanning problems. For multi-site studies however, rescan data may not available. Applying the presented method to source-target images of different subjects is unlikely to work well, since the subjects' anatomy will be too different for many voxels to even have a corresponding voxel in the other image (when looking at all features). Investigating how to obtain an FST from images of different subjects would therefore be an interesting direction for further research.

## Footnotes

1 http://www.hippocampal-protocol.net/

2 The ADNI was launched in 2003 as a public-private partnership, led by Principal Investigator Michael W. Weiner, MD. The primary goal of ADNI has been to test whether serial magnetic resonance imaging (MRI), positron emission tomography (PET), other biological markers, and clinical and neuropsychological assessment can be combined to measure the progression of mild cognitive impairment (MCI) and early Alzheimer's disease (AD). For up-to-date information, see http://www.adni-info.org.

3 http://www.adni.loni.usc.edu

4 http://crl.med.harvard.edu/software/CRKIT/index.php

5 We used version 1.3, without corrective learning. The implementation is available at http://www.nitrc.org/frs/?group_id=634.

6 not for the FST methods, which could not be calculated because of absence of source-target image pairs between sites
